# CHADS_2_ and CHA_2_DS_2_-VASc Scoring Systems for Predicting Atrial Fibrillation following Cardiac Valve Surgery

**DOI:** 10.1371/journal.pone.0123858

**Published:** 2015-04-07

**Authors:** Liang Yin, Xinyu Ling, Yufeng Zhang, Hua Shen, Jie Min, Wang Xi, Jing Wang, Zhinong Wang

**Affiliations:** Department of Cardiothoracic Surgery, Changzheng Hospital, Second Military Medical University, Shanghai, 200003, China; Harefield Hospital, UNITED KINGDOM

## Abstract

**Objective:**

Clinical use of CHADS_2_ and CHA_2_DS_2_-VASc scoring systems for predicting AF following cardiac surgery have been reported in previous studies and demonstrated well-validated predictive value. We sought to investigate whether the two scoring systems are effective for predicting new-onset of AF following cardiac valve surgery and to demonstrate its potential utility of clinical assessment.

**Methods:**

Medical records of all patients underwent cardiac valve surgeries during the period of January 2003 and December 2013 without preoperative AF at the cardiac center of our university were reviewed. The main outcome end point of our study was the early new-onset of AF following cardiac valve surgery.

**Results:**

There were overall 518 patients involved in this study, with 234 (45.17%) developed POAF following valve surgery. Patients with POAF had older age (*P*=0.23) and higher BMI (*P*=0.013) than those without POAF. History of heart failure (*P*=0.025), hypertension (*P*=0.021), previous stroke or TIA (*P*=0.032), coronary artery disease (*P*=0.001), carotid artery disease (*P*=0.024) and preoperative medication of statins (*P*=0.021) were significantly more recorded in POAF group. Patients with POAF also had higher LAD (*P*=0.013) and E/e’ ratio (*P*<0.001). The CHADS_2_ and CHA_2_DS_2_-VASc scores were significantly higher in patients with POAF (*P*=0.002; *P*<0.001), and under univariate and multivariate regression analysis the CHADS_2_ and CHA_2_DS_2_-VASc scores were significant predictors of POAF (*P*=0.001; *P*<0.001). Based on stratification of CHADS_2_ and CHA_2_DS_2_-VASc scores, the Kaplan-Meier analysis obtained a higher POAF rate on patients with higher stratification of CHADS_2_ and CHA_2_DS_2_-VASc scores (*P*<0.001; *P*<0.001).

**Conclusion:**

In conclusion, CHADS_2_ and CHA_2_DS_2_-VASc scores were directly associated with the incidence of POAF following valve surgery and a higher score was strongly predictive of POAF.

## Introduction

Atrial fibrillation (AF) is the one of the most common complications after cardiac surgery, and postoperative atrial fibrillation (POAF) following cardiac surgery is one of the most predictors of high mortality in the long-term[1]. Although the exact cause and mechanism of POAF have not been demonstrated and testified, injury of the atrium and systematic inflammation may play important roles in this postoperative arrhythmia process [2–4]. POAF is usually well tolerated, but may result in hypotension, heart failure, stroke, longer hospital stay and increased hospital costs[5]. POAF usually occurs within the first 5 days after cardiac surgery, with a peak incidence on days 2 and 3[6]. Predictors, such as older age, number of bridge vessels in coronary artery bypass grafting (CABG) and left ventricular (LV)hypertrophy had been verified in highly incidence of POAF following cardiac surgery[7]. But only several scattered indicators and lack of systematic scoring system for predicting POAF deter the assessing process preoperatively, and increased perioperative morbidity and long-term mortality may occur in concerned patients.

Originally, the CHADS_2_ [8] [cardiac failure, hypertension, age, diabetes, stroke (doubled)] risk index, a point system in which 2 points are assigned for a history of stroke or transient ischemic attack (TIA) and 1 point each is assigned for age≥75 years, a history of hypertension, diabetes, or recent heart failure, is an easy-to-remember means of assessing stroke risk of patients with AF. CHA_2_DS_2_-VASc [9] [congestive heart failure, hypertension, age ≥75 (doubled), diabetes, stroke (doubled), vascular disease, age 65–74, and sex category (female)], a newly adjusted scheme recommended by European Society of Cardiology (ESC) is based on a point system in which 2 points are assigned for a history of stroke or TIA, or age ≥75; and 1 point each is assigned for age 65–74 years, a history of hypertension, diabetes, recent heart failure, vascular disease (myocardial infarction, complex aortic plaque, and peripheral arterial disease (PAD), including prior revascularization, amputation due to PAD, or angiographic evidence of PAD, etc.), and female sex[10–12].

The CHADS_2_ and CHA_2_DS_2_-VASc scores can provide easy and effective assessment of patients with AF in which circumstances they can occur stroke or other thrombus-embolism events[13]. Only limited studies have investigate the association between CHADS_2_ or CHA_2_DS_2_-VASc score and prediction of POAF, one of the studies[14] mentioned that assessment using CHADS_2_ and CHA_2_DS_2_-VASc scores is predictive of POAF after cardiac surgery and may be helpful for identifying high-risk patients, but differentiation on types of surgery have not been studied in this report. In this study, we sought to explore the potential association between CHADS_2_ and CHA_2_DS_2_-VASc scores and its utility of assessment for predicting POAF following cardiac valve surgery.

## Methods

### Patients

Concerning the difficulties in meeting with each patient and some patients were not convenient to be offered with written informed consent, written informed consent was not utilized in our study, permission of medical data extraction was obtained by verbal informed consent from each patient, which was recorded by telephone for each patient included in our study, and the consent procedure of our study and this study were approved by Ethics Committee of Shanghai Changzheng Hospital. This study included a series of 518 patients in our center from January 2003 and December 2013 who underwent cardiac valve surgeries (valve repair or valve replacement) without preoperative AF, whose baseline characteristics are shown in Table 1. This series belongs to a consecutive series who were carried out cardiac valve surgeries with or without CABG, non-valve surgeries were excluded because their immediate and late outcomes are likely to differ from those who were underwent valve surgeries. Patients with preoperative AF, pacemaker implantation, Cox maze or radiofrequency ablation procedure for atrial arrhythmia were also excluded from our study.

**Table 1:** Baseline characteristics of patients with and without POAF.

	POAF(n = 234)	no POAF(n = 284)	*p* value
Age	59.1±7.9	55.6±18.0	.023
Age 65–75 y	98(41.88%)	65(22.89%)	.043
Age ≥75 y	6(2.56%)	9(3.17%)	.123
Female	89(38.03%)	101(35.56%)	.216
Smoke	79(33.76%)	100(35.21%)	.065
BMI (kg/m^2^))	26.0±5.8	23.1±7.6	.013
Medical history			
Heart failure	74(31.62%)	65(22.89%)	.025
Hypertension	110(47.01%)	72(25.35%)	.021
Diabetes mellitus	43(18.38%)	30(10.56%)	.075
Stroke or TIA	15(6.14%)	7(2.46%)	.032
Coronary artery disease	60(25.64%)	35(12.32%)	.001
Carotid artery disease	41(17.52%)	31(10.92%)	.024
Peripheral arterial disease	36(15.83%)	24(8.45%)	.051
Type of surgery			
MVP	4619.66%)	59(20.77%)	.413
MVR	101(43.16%)	121(42.61%)	.876
AVR	80(34.19%)	100(35.21%)	.231
TVP	31(13.25%)	52(18.31%)	.075
TVR	3(1.28%)	3(1.06%)	.987
Concomitant CABG	49(20.94%)	18(6.34%)	.001
Preoperative medication			
ACEIs or ARBs	89(38.03%)	10235.92%)	.672
β-Blockers	97(41.45%)	121(42.61%)	.901
Calcium channel blockers	90(38.45%)	97(34.15%)	.087
Statins	74(31.62%)	72(25.35%)	.021
Aspirin	123(52.56%)	167(58.80%)	.067
Clopidogrel	41(17.53%)	65(22.89%)	.078
Echocardiographic features			
LVEF(%)	56.4±3.3	56.5±9.3	.432
LVEDD(cm)	5.4±1.7	5.0±0.7	.213
LVM(cm3)	106.8±43.7	107.3±38.7	.768
LAD(cm)	5.2±1.3	4.5±0.9	.013
E/e’ ratioratio	18.9±5.1	12.1±3.5	＜.0001
CHADS_2_ score	1.6±0.8	0.78±0.9	.002
CHA_2_DS_2_-VASc score	3.2±1.2	1.6±1.1	.＜.001

Data are presented as n (%) or mean±SD. POAF, postoperative atrial fibrillation; BMI, body mass index; TIA, transient ischemic attack; MVP, mitral valvuloplasty; MVR, mitral valve replacement; AVR, aortic valve replacement; TVP, tricuspid valvuloplasty; TVR, tricuspid valve replacement; CABG, coronary artery bypass grafting; LVEF, left ventricular ejection fraction; LVEDD, left ventricular end-diastolic diameter;LVM, left ventricular mass; LAD, left atrial diameter; ACEI, angiotensin-converting enzyme inhibitor; ARB, angiotensin receptor blockers.

CHADS_2_ and CHA_2_DS_2_-VASc scores of each patient, echocardiographic reports and complete medical records were collected to investigate the association between CHADS_2_ and CHA_2_DS_2_-VASc scores and the occurrence of POAF following valve surgery.

### CHADS_2_ and CHA_2_DS_2_-VASc Scores and Endpoint

The CHADS_2_ score was calculated for all the patients by assigning 1 point for congestive heart failure, hypertension, age ≥75, and diabetes mellitus. A further 2 points was added for the criterion of previous stroke or TIA. The expanded CHA_2_DS_2_-VASc score scheme is based on a point system in which 2 points each are assigned for age ≥75 and for history of stroke, TIA, or thromboembolism and 1 point is assigned for congestive heart failure, hypertension, diabetes mellitus, age 65~75 years, vascular disease (myocardial infarction, complex aortic plaque, and PAD, including prior revascularization, amputation due to PAD, or angiographic evidence of PAD, etc.), and female sex category[8, 15].

The main outcome endpoint of interest was new-onset POAF occurred during the 30-day postoperative period or before discharge from hospital. POAF was defined as documented AF episodes lasting longer than 30 seconds recorded by continuous telemetry throughout hospitalization or by electrocardiography within the 30-day follow-up following valve surgeries. The diagnosis of POAF was then confirmed by a cardiologist to minimize the error that may contribute to the bias generated in our study.

### Statistical analysis

Statistical analysis was carried out using SPSS 18.0 software. Quantitative data are expressed as mean±SD and were compared with 2-sample *t* tests for independent samples, whereas dichotomous variables were reported as absolute values and proportions. Differences in proportion were compared with a χ^2^ test or Fisher exact test as appropriate. Univariate analysis was performed with the Kaplan–Meier test and with the Cox regression analysis. For each variable, the odds ratio (OR), 95% confidence interval (CI), and *P* value were provided. Variables significantly associated with POAF after univariate analysis (*P*<0.05) and those that were established risk factors were entered in a multivariable logistic regression model to identify the independent predictors of POAF[16]. The POAF-free survival curves were constructed according to the Kaplan–Meier method. Additionally, we calculated the area under receiver operating characteristic (ROC) curve to assess the predictive value of CHADS_2_ and CHA_2_DS_2_-VASc scores for POAF.

## Results

### Clinical Characteristics of patients

We collected data on patient’s age, gender, medical history and type of valve surgery, laboratory and echocardiography data related to surgery were also recorded. The study population consists of 518 patients, 234 (45.17%) of which developed POAF following cardiac valve surgeries. The whole population had a mean age of 58.2±9.9 and comprised 190 (36.67%) woman and 328 (63.32%) men. 139 (26.83%) of the study patients had a history of heart failure,182 (35.13%) had hypertension, 73 (14.09%) had diabetes and 22 (4.24%) of them had a history of stroke or TIA. Vascular disease was found existed in 121 (23.35%) patients, in which 95 (18.34%) had coronary artery disease, 72 (13.90%) had carotid artery disease and 60 (11.58%) had peripheral artery disease.

The baseline characteristics of patients with POAF and without POAF were depicted in Table 1. Patients with POAF developed this incident at a median of 3.6 ±12.5 days (range, 1–21). Comparing with patients without POAF, patients with POAF had significantly higher age (*P* = 0.23) and higher body mass index (BMI) (*P* = 0.013), higher prevalence of heart failure (*P* = 0.025), hypertension (*P* = 0.021), previous stoke or TIA (*P* = 0.032), coronary artery disease (*P* = 0.001), carotid artery disease (*P* = 0.024) and preoperative medication of statins (*P* = 0.021) were also significantly more recorded in POAF group. The POAF group also had higher left atrial diameter (LAD) (*P* = 0.013) and E/e’ ratio (*P*＜0.0001). There were no significant differences in terms of left ventricular ejection fraction (LVEF) (*P* = 0.432), left ventricular end-diastolic diameter (LVEDD) (*P* = 0.213), left ventricular mass (LVM) (*P* = 0.768) between patients with and without POAF.

Type of surgery was also collected in our study. Mitral valvuloplasty (MVP) and mitral valve replacement (MVR) were performed on 105 (20.27%)and 222 (42.86%) patients respectively. Aortic valve replacement (AVR), tricuspid valvuloplasty (TVP) and tricuspid valve replacement (TVR) were carried out on 180 (34.75%), 83 (16.02%) and 6 (1.16%) patients respectively. There was no significant difference between two groups in surgery performed except that concomitant CABG were more frequently carried out on patients with POAF(*P* = 0.001).

### CHADS_2_ and CHA_2_DS_2_-VASc Scores

**Fig 1:**
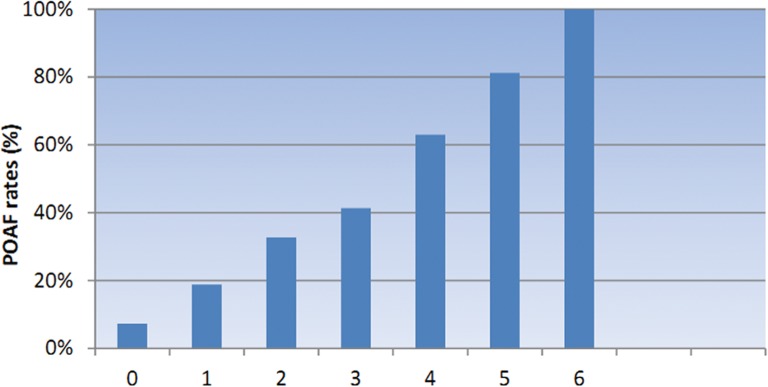
POAF rates and CHADS_2_ scores. The POAF rates are higher in greater CHADS_2_ scores.

**Fig 2:**
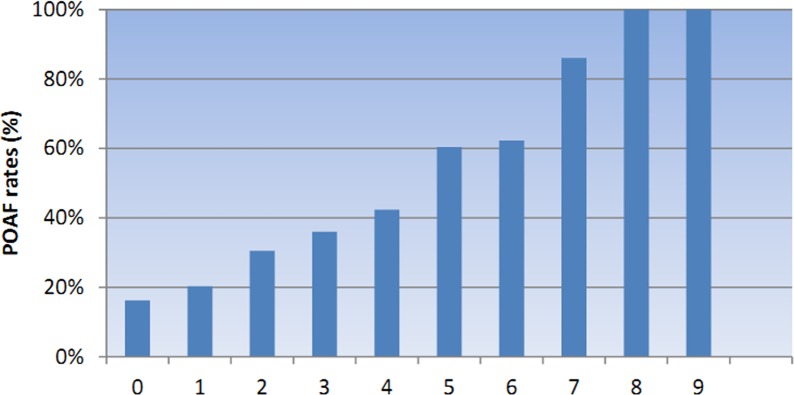
POAF rates and CHA_2_DS_2_-VASc scores. The POAF rates incrementally increased as the CHA_2_DS_2_-VASc score increased.

Data on CHADS_2_ and CHA_2_DS_2_-VASc scores of patients were presented in Table 1. Patients who developed POAF after surgery had significant higher CHADS_2_ and CHA_2_DS_2_-VASc scores than those without POAF (*P* = 0.002; *P*＜0.001), and the univariate and multivariate regression analysis showed that the CHADS_2_ and CHA_2_DS_2_-VASc scores were significant predictors of POAF following valve surgery (*P* = 0.002; *P*＜0.001)(Table 2 and 3). As shown in Figs 1 and 2, the incidence rate of POAF was strongly associated with CHADS_2_ and CHA_2_DS_2_-VASc scores. The incidence of POAF incrementally increased with CHA_2_DS_2_-VASc scores, and higher CHADS_2_ scores was predictive of higher POAF incidence rate.

**Table 2:** Univariate regression analysis for predictors of POAF.

Variable	Odds ratio	95%CI	*p* value
Age(y)	0.952	0.880–1.013	.021
BMI (kg/m^2^)	1.562	1.167–3.087	.014
LAD (cm)	1.906	0.986–2.034	.035
LVM(cm^3^)	1.032	0.984–1.321	.543
LVEF(%)	1.057	0.985–1.521	.342
E/e’ ratio	1.310	1.011–1.426	.041
Smoke	1.567	0.555–3.876	.871
Diabetes	7.089	1.980–30.187	.104
Hypertention	1.890	0.560–13.564	.453
Heart failure	1.123	1.012–2.539	.024
CHADS_2_ score	1.578	1.101–2.314	.001
CHA_2_DS_2_-VASc score	1.405	1.032–2.004	＜.001

CI, Confidence interval; POAF, postoperative atrial fibrillation; BMI, body mass index; LAD, left atrial diameter; LVM, left ventricular mass;LVEF, left ventricular ejection fraction; ACEI, angiotensin-converting enzyme inhibitor;ARB, angiotensin receptor blockers.

**Table 3:** Multivariate regression analysis for predictors of POAF with CHADS_2_ and CHA_2_DS_2_-VASc score.

Variable	Odds ratio	95%CI	*p* value
With CHADS_2_ score			
Age(y)	0.879	0.566–1.043	.041
BMI (kg/m2)	0.405	0.306–0.889	.001
LAD (cm)	0.323	0.100–0.585	.016
E/e’ ratio	0.906	0.813–1.214	.694
Heart failure	0.678	0.311–0.990	.032
CHADS_2_ score	0.411	0.210–0.608	＜.001
With CHA_2_DS_2_-VASc score			
Age(y)	0.899	0.654–1.005	0.033
BMI (kg/m2)	0.443	0.223–0.754	.001
LAD (cm)	0.651	0.443–0.938	.021
E/e’ ratio	0.515	0.331–1.721	.053
Heart failure	0.770	0.556–1.743	.041
CHA_2_DS_2_-VASc score	0.577	0.436–0.889	.002

CI, Confidence interval; POAF, postoperative atrial fibrillation; BMI, body mass index; LAD, left atrial diameter; LVEF, left ventricular ejection fraction.

**Fig 3:**
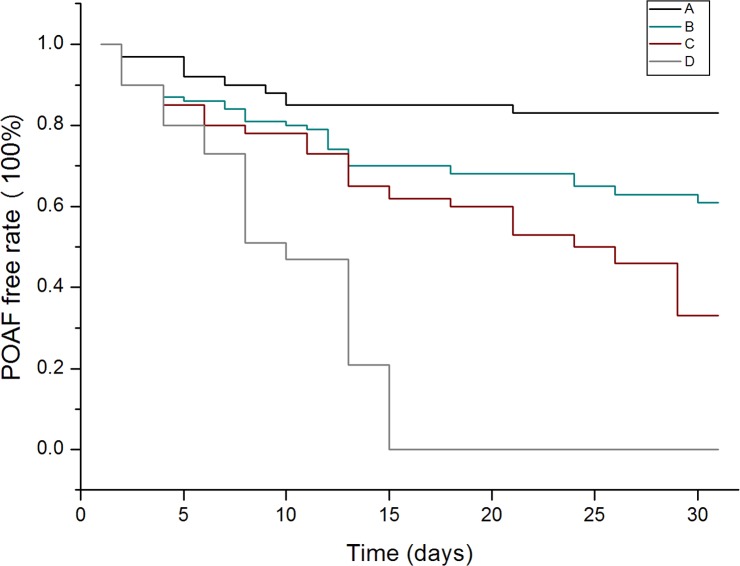
POAF-free rate curves for patients with CHADS_2_ score. The Kaplan-Meier survival analysis showed that patients with higher stratification of CHADS_2_ score had a higher event rate than those who had lower stratification of CHADS_2_ score. (*P* = 0.002). A-(0–1 point), B- (2–3 points), C-(4–5 points), D-(6 points).

**Fig 4:**
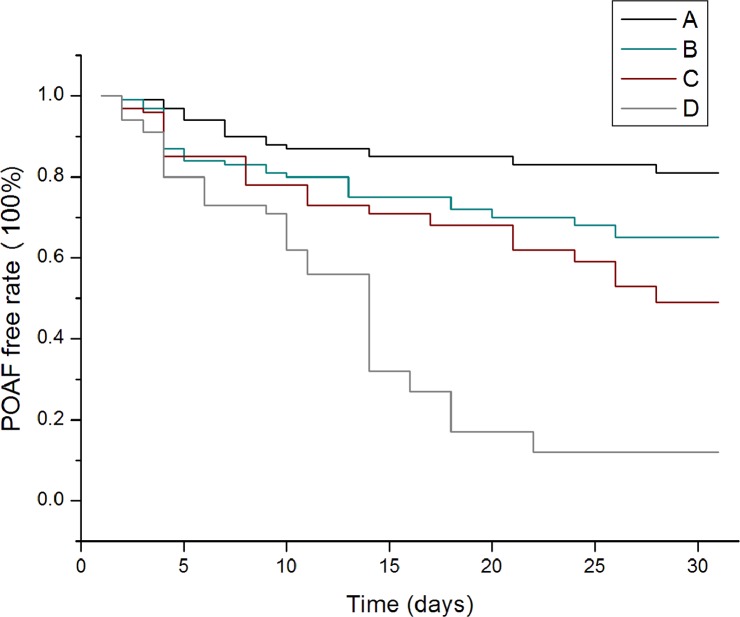
POAF-free rate curves for patients with CHA_2_DS_2_-VASc score. The Kaplan-Meier survival analysis showed that patients with higher stratification of CHA_2_DS_2_-VASc score had a higher event rate than those who had lower stratification of CHA_2_DS_2_-VASc score. (P = 0.037). A-(0–1 point), B- (2–3 points), C-(4–5 points), D-(≥6 points).

In the Kaplan-Meier analysis, we stratified CHADS_2_ and CHA_2_DS_2_-VASc scores. In CHADS_2_ score, we categorized score 0–1 as A, score 2–3 as B, score 4–5 as C and score 6 as D, while in CHA_2_DS_2_-VASc score we categorized score 0–1 as A, score 2–3 as B, score 4–5 as C and score greater than 6 as D. As demonstrated in *Figs 3*and *4*, the cumulative event rates of POAF in Kaplan-Meier survival curves showed that patients with higher stratification of CHADS_2_ and CHA_2_DS_2_-VASc scores had a significant higher incidence rate of POAF (*P* = 0.001; *P* = 0.011).

**Fig 5:**
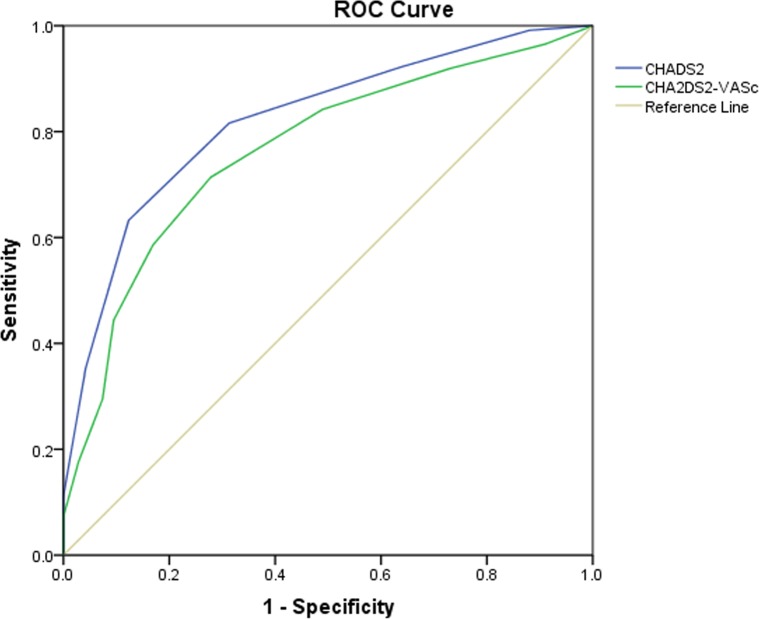
Predictive value of CHADS_2_ and CHA_2_DS_2_-VASc scores for incidence of POAF following valve surgery under ROC. The area under ROC was 0.821 (95%CI, 0.784–0.857, P＜0.001) and 0.765 (95%CI, 0.723–0.807, P＜0.001) respectively.

The predictive value of CHADS_2_ and CHA_2_DS_2_-VASc scores for incidence of POAF following valve surgery were similar, as depicted in *Fig 5,* the area under ROC was 0.821 (95%CI, 0.784–0.857, *P*＜0.001) and 0.765 (95%CI, 0.723–0.807, *P*＜0.001) respectively.

## Discussion

POAF is associated with increased risk of thromboembolism and stroke, and POAF following cardiac surgery is one of the major risk factors affecting long-term morbidity and mortality of patients[17]. Therefore, the assessment of risk factors and predictors preoperatively are important for decision-making regarding the use of thromboprophylaxis. Age, gender, hypertension, diabetes mellitus, and LV function have been reported as predictors preoperatively for POAF following cardiac surgery[18–20], but to date there is no published systematic scheme involving these predictors to provide a scoring scheme under which POAF can be evaluated and predicted. In a study conducted by Su-Kiat Chua[14], he and his colleagues initially explored the relationship between CHADS_2_ and CHA_2_DS_2_-VASc scores and POAF following cardiac surgery, they demonstrated that CHADS_2_ and CHA_2_DS_2_-VASc scores were predictive of POAF after cardiac surgery and may be helpful for identifying high-risk patients. But to the differentiation of incidence of POAF according to type of surgery, there is limitation using this scheme. It is testified that valve procedures is associated with an increased incidence of POAF compared with coronary artery disease surgeries [21]. Therefore, in this study, we sought to identify the potential utility of CHADS_2_ and CHA_2_DS_2_-VASc scores system preoperatively in assessment of POAF following heart valve surgery.

A cohort of 518 patients were included in this study who had no history of preoperative AF underwent valve surgeries, and most of the enrolled patients were underwent valvuloplasty and valve replacement. The whole incidence of POAF following valve surgery was 45.17% in this study, which was similar to previous reported studies[22]. As shown in the aforementioned results, we found that the incidence rate of POAF following valve surgery incrementally increased with increased CHADS_2_ or CHA_2_DS_2_-VASc scores, and the incidence of POAF between individual types of valve surgery showed no significant difference. Furthermore, patients with CHADS_2_ or CHA_2_DS_2_-VASc scores at higher stratification had a significant higher occurrence of AF than those who had lower stratification. We also calculated the individual occurrence of POAF following valve surgery based on different points from naught to the highest. As demonstrated in Figs 1 and 2, point of 2 in CHADS_2_ and 4 in CHA_2_DS_2_-VASc were turning points where the incidence of POAF increased significantly than patients with lower CHADS_2_ or CHA_2_DS_2_-VASc score. Furthermore, in our multivariable logistic regression analysis, the CHADS_2_ and CHA_2_DS_2_-VASc scores were valuable predictors for POAF following valve surgeries.

The CHADS_2_ and CHA_2_DS_2_-VASc scores are accurate and are both easily and widely used in prediction of severity and outcome of stroke, providing decision-making management in patients with AF[12, 23]. In this study, we found that the CHADS_2_ and CHA_2_DS_2_-VASc scores are also helpful in predicting POAF following valve surgery. Based on such a hypothesis, we recommend its extended clinical trial and popularized its potential utility clinically in patients underwent cardiac surgeries. Conducted preoperatively using CHADS_2_ and CHA_2_DS_2_-VASc scores, we may provide primary impression of occurrence of POAF in patients underwent valve surgery, which can be helpful in the prophylactic medication use to minimize the opportunity of onset of POAF and also reduce the complications related to POAF.

As demonstrated in previous studies, an incremental deterioration of left ventricle (LV) diastolic dysfunction may cause left atrium (LA) enlargement, which is strongly associated with the occurrence of POAF, and whose underlying mechanism may due to LA strain vulnerability[24]. In our study, the LAD and E/e’ ratio of patients who had POAF were significantly higher than those without POAF, which verified the above view of points. Congestive heart failure, one of the most important components in CHADS_2_ and CHA_2_DS_2_-VASc scores system, is morbid condition that shares common risk factors and frequently coexisted with AF. Patients with heart failure usually showed a decreased LV function, especially in those with advanced ages. Furthermore, the unstable hemodynamic status of AF patients also contribute to deteriorated heart failure, each condition predisposes to the other. However, LVEF, LVEDD and LVM showed no significant differences between two groups in this study. Hypertension, diabetes, and vascular disease, another several components of CHADS_2_ and CHA_2_DS_2_-VASc scores system, frequently influencing the LA diastolic function, also can promote the incidence of POAF after valve surgery.

The main result of this study is the predictive ability of these two risk scores systems for POAF in patients without preoperative AF. The outcome indicates that the CHADS_2_ and CHA_2_DS_2_-VASc scores can be used as an effective tool for predicting the incidence of POAF following valve surgery. Furthermore, the primary findings of our study resemble the results of previous published reports which demonstrated the predictors of POAF following cardiac surgery. We creatively combined the CHADS_2_ and CHA_2_DS_2_-VASc scores with occurrence of POAF following valve surgery to investigate the potential relationship between them, and the result showed that the incidence rate of POAF following valve surgery is strongly associated with higher CHADS_2_ and CHA_2_DS_2_-VASc scores. This relationship may be helpful to discriminate patients who were predisposed to high POAF occurrence, and which may also provide us decision-making strategies of preoperative prophylactic medication. We recommended that high risk patients with a CHADS_2_ score greater than 2 and CHA_2_DS_2_-VASc score greater than 4 may benefit from prophylactic use of β-block and those antiarrhythmic agents before cardiac surgery[25]. We also observed that the CHADS_2_ and CHA_2_DS_2_-VASc scores are also predictive of late all-cause mortality, which demonstrates a possible wider clinical utility of these scores.

The main limitation of our study is its retrospective design. Also, we evaluated the early onset of POAF following valve surgery alone without comparison with those underwent other types of cardiac surgery. And the follow-up period of patients were not investigated in this study. Nevertheless, our study provides useful information related to the risk factors of POAF. A larger clinical trial is necessary to assess the underlying usefulness of CHADS_2_ and CHA_2_DS_2_-VASc scores in predicting POAF to assure whose clinical utility.
